# Using Smartphone Sensor Paradata and Personalized Machine Learning Models to Infer Participants’ Well-being: Ecological Momentary Assessment

**DOI:** 10.2196/34015

**Published:** 2022-04-28

**Authors:** Alexander Hart, Dorota Reis, Elisabeth Prestele, Nicholas C Jacobson

**Affiliations:** 1 Research Group Applied Statistical Modeling Department of Psychology Saarland University Saarbrücken Germany; 2 Research Group Diagnostics, Differential and Personality Psychology, Methods and Evaluation Department of Psychology University of Koblenz-Landau Landau Germany; 3 Center for Technology and Behavioral Health, Departments of Biomedical Data Science and Psychiatry Geisel School of Medicine Dartmouth College Lebanon, NH United States

**Keywords:** digital biomarkers, machine learning, ecological momentary assessment, smartphone sensors, internal states, paradata, accelerometer, gyroscope, mood, mobile phone

## Abstract

**Background:**

Sensors embedded in smartphones allow for the passive momentary quantification of people’s states in the context of their daily lives in real time. Such data could be useful for alleviating the burden of ecological momentary assessments and increasing utility in clinical assessments. Despite existing research on using passive sensor data to assess participants’ moment-to-moment states and activity levels, only limited research has investigated temporally linking sensor assessment and self-reported assessment to further integrate the 2 methodologies.

**Objective:**

We investigated whether sparse movement-related sensor data can be used to train machine learning models that are able to infer states of individuals’ work-related rumination, fatigue, mood, arousal, life engagement, and sleep quality. Sensor data were only collected while the participants filled out the questionnaires on their smartphones.

**Methods:**

We trained personalized machine learning models on data from employees (N=158) who participated in a 3-week ecological momentary assessment study.

**Results:**

The results suggested that passive smartphone sensor data paired with personalized machine learning models can be used to infer individuals’ self-reported states at later measurement occasions. The mean *R*^2^ was approximately 0.31 (SD 0.29), and more than half of the participants (119/158, 75.3%) had an *R*^2^ of ≥0.18. Accuracy was only slightly attenuated compared with earlier studies and ranged from 38.41% to 51.38%.

**Conclusions:**

Personalized machine learning models and temporally linked passive sensing data have the capability to infer a sizable proportion of variance in individuals’ daily self-reported states. Further research is needed to investigate factors that affect the accuracy and reliability of the inference.

## Introduction

### Background

In mental health care, learning about the trajectories of a patient’s psychological strain often requires repeated verbal interactions. Interviewing the patient in person is not always preferable because of the economic burden posed on clinicians and patients alike. A substitute for these interviews are questionnaires that patients answer in pen and paper diaries or on their mobile devices. Although these methods allow for a relatively cheap and fine-grained examination of the participants’ condition, responding multiple times to the same questionnaire can still be tiring and burdensome for patients, leading to increased noncompliance. In the last decade, biomarkers from mobile sensors have emerged as a promising alternative to infer patients’ psychological conditions (eg, depression severity). However, thus far, many of the proposed methods incidentally collect plenty of unrelated private data, require specialized apps on the patients’ devices, or depend on the environment (eg, cell phone signal strength). Inferring a patient’s condition from movement sensors commonly available in smartphones and recorded only while participants fill out a questionnaire could be an alternative that has not been explored so far. Therefore, in this study, we investigated the predictive capabilities of such sparse data collected during an unrelated web-based study. Although this method initially relies on questioning the patient via identical web-based questionnaires, participant burden could be attenuated in the long run by rotating questionnaire topics or omitting parts of the questionnaire.

### Theoretical Background

In the last 40 years, our understanding of fluctuations and trajectories of psychological characteristics has benefited greatly from the adoption of ecological momentary assessment (EMA). EMA studies typically focus on assessing a person’s state at a set of moments throughout the day. This schedule is then repeated over a number of days of interest (see Shiffman et al [1] for a thorough introduction to the methodology). EMA moves the assessment closer to the real-life occurrence of relevant phenomena. For example, it allows for the recording of mood states before medication is misused [2]. This fine-grained assessment allows investigations to be conducted on the interplay between variables in everyday life while diminishing memory biases [3]. Using EMA across a considerable length of time consequently allows an investigator to inspect and monitor the course of an individual’s responses without requiring direct interaction between the participant and investigator. Consequently, EMA could be a fruitful tool in researchers’ and practitioners’ toolsets as it can be used to discover more about the moment-to-moment changes that are taking place in a person’s condition. However, answering a sizable number of questions several times a day over many days can also be disruptive and time-consuming for EMA participants. Therefore, concerns have been raised about whether the burden it imposes on the individual may even undermine the effort of collecting ecologically valid data [1,4].

Reducing burden in general is of fundamental concern for research ethics [5] and, thus, in the long run, it is of concern for patients and practitioners using frequently repeated interviews. Participant attrition, which can be an outcome of burden, has been highlighted as a major threat to validity in eHealth research [6,7]. In the case of EMA methodology, increased burden has also been linked to reduced data quantity and quality [8,9]. Reasons for the additional burden may vary from study to study, but sampling frequency and number of items per prompt have repeatedly been identified as relevant factors [9-13]. Consequently, a common strategy for reducing burden in EMA studies is to shorten the questionnaire [9,14]. At the same time, omitting items from a questionnaire can negatively affect the reliability or validity of the measurement. This poses a dilemma for using EMA to its full potential in research and clinical application.

A way to overcome this limitation is to passively collect behavioral data. With behavioral data, one could attempt to infer participants’ states without requiring them to explicitly report their symptoms. Smart devices, which can monitor their users’ behavior in multiple ways [15], enable researchers to gather, explore, and leverage such data. Today, many field studies using EMA or comparable methodologies are already using smartphones for assessment [13,16]. There have also been several successful attempts to identify behavioral information that indicates psychological characteristics, often termed digital biomarkers or behavioral markers, which comprise the field of personal sensing [17]. Scholarly articles on the exploration of passively collected data range from inferring participants’ traits from their smartphone use [18-20] to inferring momentary expressions [21]. As the field is quite young, it still comprises a variety of techniques, operationalizations, and outcomes and requires more evidence regarding the effectiveness of approaches and measurement validity (see Mohr et al [17] for an overview pertaining to mental health). Focusing on the areas of mental health and well-being, Yim et al [22] recently reviewed studies that explicitly conducted personal sensing alongside smartphone-based EMA or substituted passive sensing for EMA in the context of major depression. Most studies successfully identified participants with depressive symptoms and inferred their stress levels or their levels of (negative) emotion. Consequently, passive sensing appears to be a fruitful option to overcome burden by passively collecting information from participant biomarkers. At the same time, the studies reviewed by Yim et al [22] showed considerable heterogeneity in terms of the sensor data, how the data were collected, and which methods were used to analyze the data.

### Open Challenges for Alleviating the EMA Burden With Personal Sensing

Many passive sensing approaches seem to work equally well for inferring states related to mental health. However, the heterogeneity in these approaches leaves unclear which methodological choices should inform a reliable and applicable approach that could alleviate burden in EMA surveys. For example, Mohr et al [17] identified open challenges pertaining to study quality, reproducibility, variability, uncertainty, and privacy. This investigation addressed 3 of these major challenges.

First, passive sensing studies have shown a distinct lack of agreement on the implemented validation strategies. At the same time, choosing an inappropriate validation strategy for the intended application threatens the validity of the inference. In this vein, Saeb et al [23] investigated the cross-validation scheme of 64 passive sensing studies that aimed to infer clinical outcomes. They found that 45% of the studies used a cross-validation that overestimated the capabilities of the models. Hence, choosing a correct training and cross-validation method is of special concern when data from passive sensing will be used to infer unobserved characteristics of the participants. One of the most popular methods in the studies reviewed by Yim et al [22] and Saeb et al [23] is training and cross-validating the models on a random subset of the entire sample. However, Saeb et al [23] noted that personalized models, in which an individual’s past relationships between indicators and states are used to derive models that can infer future states based on future indicator data, might be more appropriate. The viability of such personalized models has only been explored in a few studies related to EMA [24-26]. For example, Asselbergs et al [24] collected the self-reported mood of 27 participants at 5 time points each day over the course of 6 weeks. In addition, the authors unobtrusively logged information about phone calls, SMS text messages, screen activation, app use, and camera use. Asselbergs et al [24] used these data to compute personalized models to infer each participant’s mood using forward stepwise regression. Within an error margin of 0.5 around the observed scores, the models inferred between 55% and 76% of the responses on average. However, the authors were not able to replicate the rate of 93% that had been presented in an earlier study [27]. As previous research has not explored which validation strategy can be deemed most appropriate for the use case of substituting EMA responses with collected sensor data, our study compared the most common approaches of training and cross-validating models across the entire sample to computing personalized models for each individual. Furthermore, we contrasted the performance of 2 popular machine learning algorithms that are common in passive sensing studies.

A second challenge in determining an appropriate method to alleviate EMA burden is the variety of available sensors and other use data. This confronts researchers with countless degrees of freedom in how features are computed and modeled [17]. Mixing sensors with distinct characteristics (eg, device orientation and geolocation) might underestimate the importance of one feature in favor of the other and, thus, lead to a biased evaluation of the predictive capabilities of a sensor in a certain setting. This mixing and matching of sensors may further lead to an unwanted interdependence of sensor readings and person and environmental characteristics [17]. Cell phone or Wi-Fi signal strength might not be the same indicator between a city and a rural area, and battery capacity as an indicator might be confounded with participants’ choice of smartphone. Furthermore, Bähr et al [28] raised concerns about several quality issues related to geolocation data. Unfortunately, geolocation data have been a favorite for inferring depressive symptoms. Underestimating the external factors influencing these data may lead to failed replication when transitioning from small-scale validation studies to studies with larger and more diverse samples. For example, Saeb et al [29] were able to train classifiers to infer the participants’ (N=208) semantic location (eg, at home, at friends’ place, or at a restaurant) from sensor data but then could not find a substantial connection to self-reported depressive symptoms. Besides the possibility that there may be no such connection, the authors partially attributed this discrepancy from previous literature to sampling from the broader American population instead of relying on samples that had been restricted to a single location (eg, available students). Such interdependencies between sample and sensor readings must be considered when choosing sensors that may be capable of being substituted for or used to complement self-reported data in a wide variety of studies. To avoid such issues, one could also restrict the investigation to a set of sensors that are for the most part independent from environmental interference. Therefore, in this investigation, we chose data from sensors that detect device movement, which are common in smart devices.

A third challenge in substituting EMA responses with sensor data is that the chosen method must not coincidentally inflict burden in any other way. Most studies investigating mobile sensing have collected and combined considerable quantities of data from various sensors throughout the day. This leads to significant requirements regarding storage and processing capabilities on the side of the investigators and might inflict considerable battery drain and unresponsiveness on the participant’s device [30]. Furthermore, collecting large quantities of sensor data may encompass information that is inherently personally identifiable (eg, names of other connected devices) or that is able to reveal sensitive details about a person (eg, geolocation) [30]. This level of detail is not necessarily needed to infer a mental state. For example, a feature Saeb et al [31] used to infer depression was the distance covered by participants, not the participants’ actual geolocations. However, once data with such detail and semantic information have been collected for a person, the data can be used to reconstruct a large portion of the person’s daily habits without the need for elaborate analysis methods [32]. At the same time, practitioners and participants might overestimate the informational security of software [33]. Participants might further dismiss privacy concerns about activity data as part of a boundary management strategy [34,35]. If the requirements for a certain health condition or the study incentives outweigh their concerns, participants might agree to data collection that they would not have consented to under other circumstances. Therefore, modern methods should strive to maximize participant privacy at a technical level. Ideally, data collection methods will ensure this data sparsity without sacrificing predictive performance or potential treatment efficacy [36,37]. Therefore, in our investigation, we chose to restrict the data collection to the times when participants were responding to their EMA questionnaires.

**This Study: Concurrent Sensing**

Our approach for tackling the aforementioned challenges involved restricting sensor dependencies and narrowing measurement time to a required minimum. Even with such sparse data, personalized models could be fit to a participant’s peculiarities within a relatively short amount of time and accurately infer future self-reports only from future sensor data.

We chose 2 sensors that are widespread in smartphones: the acceleration and orientation sensors [38]. These 2 sensors track how participants move and rotate their devices. As sensor readings are device-centric, they are mostly independent of environmental factors such as signal strength. Although accelerometers and gyroscopes are part of the sensor ensemble of many studies, it is rather uncommon for them to be used as the sole source for studies pertaining to EMA. Nevertheless, previous research has demonstrated that they can be suitable for detecting participants’ conditions. For example, wireless accelerometers have been used in medical applications to monitor Parkinson disease [39]. Data from acceleration and orientation sensors have also allowed researchers to infer participants’ emotions [40,41]. Furthermore, Kern et al [42] showed that the accelerometer data recorded alongside the survey provided information about survey completion conditions (eg, whether a participant moved while taking the survey). Well-being has also historically been linked to posture [43]. In this regard, Kuhlmann et al [44] explored the possibility of inferring momentary subjective well-being from smartphone tilt in a recent preprint and were at least partially successful. Many more studies have shown that movement-related data can be used to infer information about the device holder, from location to personality traits, thus underscoring the predictive capabilities of such data. Summarizing several of these studies, Kröger et al [45] recently even voiced privacy concerns about the accelerometer. Consequently, also for these 2 sensors, there is a need for solutions that allow sensor data to be collected more sparsely while retaining meaningful predictive capabilities.

A solution to this problem is to collect data from sensors only while participants fill out the EMA questionnaire. Data collected in this way are usually referred to as the paradata of the survey. According to Kreuter [46], paradata are behavioral by-products of computer-assisted data collection that are often neglected in studies. In the past, they have primarily been used to reduce survey error within the total survey error framework [47]; for example, by uncovering participants’ insecurity while responding to particular survey items [48]. In the same manner that paradata can be collected from web surveys, it is possible to record sensor readings directly from participants’ web browsers without relying on additional applications. Such data are naturally restricted to the moments when participants interact with the survey. In this way, readings between time points always correspond to a similar task and are therefore somewhat standardized. This implicit standardization could be helpful for reducing heterogeneity in the data. While completing the survey, participants inevitably move their phones in a particular way. Thus, it is likely that sensor data collected from moment to moment also hold a relevant amount of information that is usually covered up by continuously recorded data. Fluctuations between time points may be more informative than features extracted from sensor readings throughout the day. For example, the change in mean acceleration along a spatial axis while completing the questionnaire might be indicative of increased arousal during that particular measurement period. This subtle information might be discarded in comparison with stronger signals such as acceleration from walking during the entire day.

In our study, we explored such sensor paradata as a solution for alleviating burden in EMA methodology. We collected sensor readings from the accelerometer and gyroscope alongside an unrelated EMA study. Participants in this study were 158 employees who were recruited to report on factors pertaining to their work-related stress and after-work detachment 6 days a week for 3 weeks. We then used those data to train machine learning models on the first 13 mornings of the data collection period to infer all of a participant’s self-reported states on the last 5 mornings based on the sensor data from these days. Furthermore, this study contrasted personalized models with between-participant models for the purpose of inferring states. Personalized (or idiographically weighted) models have already been demonstrated to be feasible solutions [23-26]. However, there is no evidence about whether between-participant machine learning models might not also be a feasible or better solution when using movement sensor paradata. Finally, we inspected the results of the algorithms regarding commonalities between personalized models that may lead to a comprehensible interpretation of how characteristics of movement are related to successful inferences. Taken together, this study aimed to answer the following open questions pertaining to substituting privacy-friendly personal sensing for EMA responses: (1) Can future self-reported states be accurately inferred from sparse accelerometer and gyroscope paradata by models trained on past data? (2) Do between-person and personalized models perform differently when inferring such self-reports? (3) Are there sensor features that are particularly suitable for inferring states?

## Methods

### Recruitment

The data for this investigation were collected as part of a research project by Reis and Prestele [49]. In this project, 158 employees from different professions voluntarily participated in an experience sampling assessment for 3 weeks. The mean age of the sample was 41.6 (SD 10.9) years, and 67.1% (106/158) of the participants were women. Most of the participants (125/158, 79.1%) worked >36 hours per week. The participants were further incentivized to complete ≥50% of the measurement points with a compensation of €30 (US $34.13) or the opportunity to access 3 weeks of web-based mindfulness stress reduction training subsequent to the end of the study.

### Data Collection and Procedure

After providing informed consent and completing an intake survey, the participants began the experience sampling procedure. For each workday during the 3 weeks, the participants were prompted in the morning, directly after work, and in the evening to fill out a short questionnaire. On Saturdays, they were asked to fill out only the morning questionnaire. The content of the questionnaire varied throughout the day. Further information about the research project by Reis and Prestele [49] is provided in the publication and in the corresponding repository.

Owing to the time-varying nature of the study’s measurement points, we had to choose a subset of constructs that we would try to infer from the sensor data. We decided to omit constructs that were exclusively related to the participants’ judgments of their workplace experiences. The remaining constructs were sleep quality, life engagement, work-related rumination, fatigue, and mood (with 2 subscales). Fatigue and mood were assessed at each measurement occasion, whereas the other 3 constructs were only assessed in the morning. Using all measurement occasions for those 2 predictors would have made them incomparable with the other 3 constructs because of a much larger data corpus (48 instead of 18 measurements) and would have required the inference of mixed trajectories (from morning to evening and from day to day instead of from day to day only). Consequently, we decided to also restrict our mood and fatigue subsample to the morning measurement occasions. This resulted in 18 measurements for mood, fatigue, and sleep quality and 15 measurements for the remaining outcomes as those were not asked about on Mondays. Although all constructs were intended to be predictors, mediators, or outcomes for the model in the initial study, this paper refers to all of them as predicted outcomes of the sensor-related models in this study.

**Figure 1:**
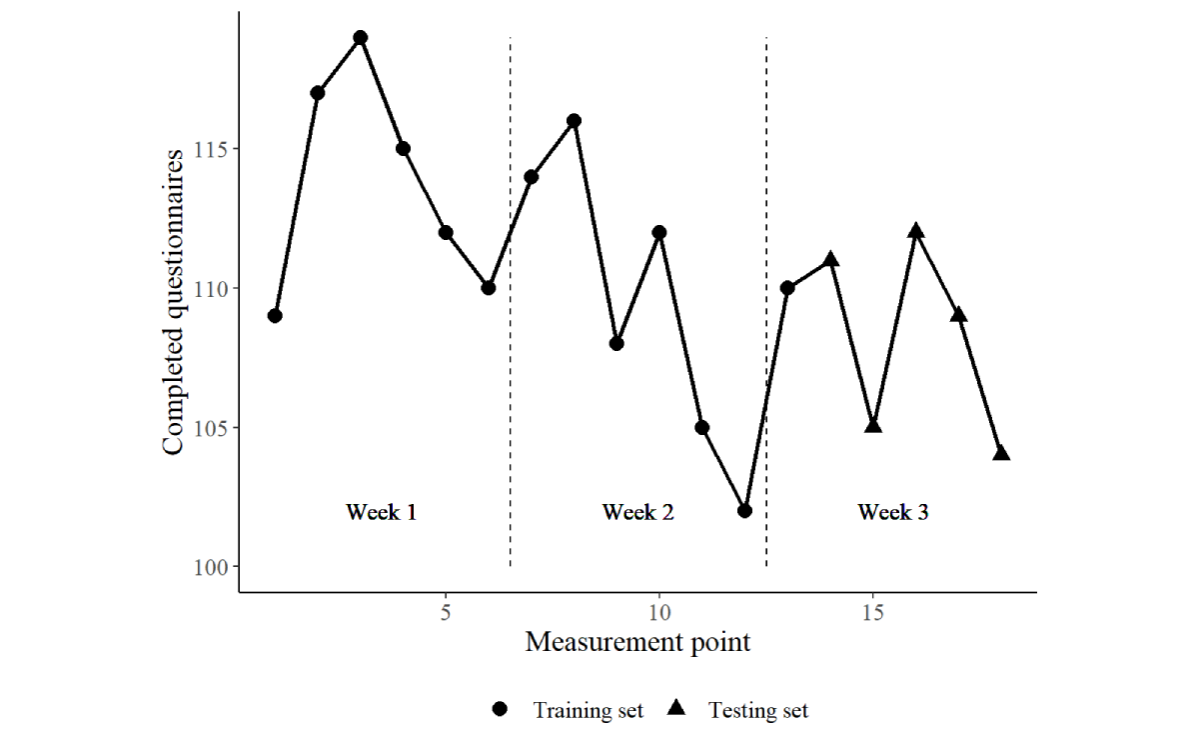
Number of questionnaires completed during the 3 weeks of assessment. Adherence varied throughout the weeks, with a declining trend toward the end of the assessment.

Sensor data were acquired by assessing the JavaScript device orientation application programming interface of the web browser at 2 Hz while the participants filled out the questionnaire, which provided data for the acceleration of the device on 3 axes and the tilting of the device at 3 angles. Although the questionnaires could be completed on a website on any computer, the participants were encouraged to use smartphones, and the questionnaires were optimized for presentation on smaller screens. Most participants complied with this recommendation, resulting in 1995 processable sensor streams out of 2204 measurements, already excluding missing or irregular data (eg, streams with an SD of 0, indicating measurement failure). Overall, adherence in this subsample was comparable between measurement occasions (Figure 1), with a visible decline over the course of the assessment. Nevertheless, the participants completed 15 out of 18 surveys on average (mean 15.11, SD 4.06). On all measurement occasions, between 65% and 75% of the questionnaires were completed, which is on par with previous EMA studies [13]. Over the course of the assessment, there was a noticeable decline in compliance common to EMA studies [9]. The start of the study and Saturdays appeared to be the least favored days by the participants. Consequently, we concluded that compliance in our sample was comparable with that reported in earlier EMA studies.

### Measures

#### Overview

As the sensor paradata for this study were collected alongside an EMA study, we were restricted to scales that the authors had chosen for investigating their hypotheses. All the self-reported measures have been validated and are commonly used in occupational health psychology. For further information regarding the underlying research project, please refer to the article by Reis and Prestele [49].

#### Fatigue

Momentary fatigue was measured on a 5-point rating scale ranging from 1 (not at all) to 5 (extremely) using a subset of 4 items from the Profiles of Mood State [50].

#### Mood and Arousal

Mood and arousal were assessed on 6 bipolar adjective pairs of the Multidimensional Mood State Questionnaire (Mehrdimensionaler Befindlichkeitsfragebogen) [51]. The participants expressed their momentary mood on a 6-point scale regarding good mood (feeling well, good, satisfied, or happy) and tense arousal (feeling tense or restless).

#### Life Engagement

Life engagement was measured using the Utrecht General Engagement Scale–3 [52], a shortened version of the Utrecht General Engagement Scale [53], which is a generalized version of the Utrecht Work Engagement Scale [54,55], which inquires about vigor, dedication, and absorption with a single item per dimension. The participants were asked to report the levels of life engagement they had experienced the previous evening on a 5-point rating scale ranging from 1 (not at all) to 5 (extremely).

#### Rumination

Work-related rumination was assessed with a selection from the items by Flaxman et al [56] (*I worried about things I need to do at work, I worried about how I would deal with a work task or issue, and my thoughts kept returning to a stressful situation at work*) that were adapted for state use from the perseverative cognition scale [57]. The participants reported the levels of rumination they had experienced the previous evening on a 5-point rating scale ranging from 1 (not at all) to 5 (extremely).

#### Sleep Quality

The sleep quality of the previous night was assessed using the sleep quality subscale of the Standardized Sleep Inventory [58]. The participants rated three adjectives (good, undisturbed, and ample) on a 5-point agreement scale ranging from 1 (not at all) to 5 (very much).

#### Extracted Features

To extract features based on the sensor data [17], we followed the recommendations of Hoogendoorn and Funk [59], who proposed that information should be aggregated within participants by computing descriptives such as central tendency, range, and variability. These recommendations were targeted toward working with self-tracking data from wearable activity trackers, thus resembling our approach of recording part of the participants’ movements while they filled out the questionnaires. For each measurement occasion, we computed the mean, the maximum of the absolute readings, the SD, the root mean square of successive differences, and the SD of successive differences across all measurements. In addition, we computed autocorrelations and partial autocorrelations up to a lag of 15. In our case, autocorrelation features imply that the device was moved or rotated similarly in a periodic manner (eg, a lag-6 autocorrelation on the z-axis would imply that the smartphone was similarly accelerated along the z-axis every 3 seconds). We also extracted the 2 highest-power frequencies from a Fourier analysis as an additional feature of periodicity in the data.

### Statistical Analysis

To investigate relationships between sensor data and outcomes, the sensor streams were arranged to follow the order of the scales during the morning assessment, thereby constructing a continuous sensor stream across all pages of the questionnaire. We then extracted features from each stream for every measurement occasion, and these features served as predictor variables for the later models. The median length of a sensor recording period across participants and measurement occasions was 68.00 seconds (5th percentile=35.00 seconds; 95th percentile=171.00 seconds) if the measurement occasion included life engagement and rumination and 45.00 seconds (5th percentile=21.20 seconds; 95th percentile=107.00 seconds) on days where only fatigue, mood, and arousal were assessed. Next, we split the data into a training set and a testing set following a 70%/30% testing scheme along the timeline of the assessment, resulting in 1455 (mood, fatigue, and sleep) or 1119 (rumination and life engagement) cases in the training set and 543 cases in the testing set. Consequently, we used our models to infer the last 5 mornings of week 3 from learning the participants’ idiosyncrasies on all previous mornings of the assessment. The final data set consisted of 230 variables (ie, 6 outcomes and 224 predictors), which we analyzed with 2 different machine learning algorithms using the *caret* package in R (R Foundation for Statistical Computing) with a separate model for each outcome. After analyzing the complete data set, individual models were computed for each participant. We then looked into relationships between self-report characteristics and feature performance that could have determined the predictive capabilities of the models.

We evaluated model performance by inspecting the *R*^2^ values of the resulting models that inferred the participants’ states using values in the testing set. In this way, we could assess how well the inferred data points recreated the observed data. For comparability with earlier studies, we further computed the accuracy criterion by LiKamWa et al [27] and Asselbergs et al [24] for the personalized models, allowing for an error margin of 0.5 around the observed value to classify the inferred value as either correct or incorrect. Finally, we explored the relationship between the sensor features and outcomes by clustering feature importance over all outcomes by means of a latent profile analysis (LPA).

### Machine Learning Algorithms

To analyze the sensor features, we chose 2 algorithms suitable for supervised learning that have been found to be reliable for different problems: random forest [60] and a penalized general linear model (GLM) [61]. Random forests make use of decision trees, whereas the penalized GLM uses regularization to fit a linear model to the data. The implementations that we chose in R were *ranger* (random forests) and *glmnet* (least absolute shrinkage and selection operator and elastic net regularized GLM). All algorithms were trained with their default values in the *caret* package and a 10-fold cross-validation of the training set.

### Ethics Approval

The study received ethics approval from the Ethics Committee of the Department of Psychology of the University of Koblenz-Landau (145_2018). Informed consent was obtained from all individual participants.

## Results

### Model Performance

Initially, we explored the models’ capabilities to infer outcomes in the complete training sample from the set of 224 predictors. The models included the features as well as the unique participant identifier (ie, a character string corresponding to the respective case) and the measurement occasion identifier (ie, an integer corresponding to the place of the measurement within the study) to provide the algorithms with information about the relationship of the repeated measurements. These results resemble a common approach in personal sensing in which a fraction of the entire data set is used to train the models. The results for all models and outcomes are shown in Table 1.

**Table 1:** *R^2^* and the root mean square error (RMSE) for all models and outcomes using the full feature set.

Outcome		Random forest		Penalized GLM^a^	
		*R^2^*	RMSE	*R^2^*	RMSE
**Training sample**					
	Sleep quality	0.16	0.87	*0.19* ^b^	0.85
	Fatigue	0.21	0.85	*0.28*	0.80
	Good mood	0.25	0.88	*0.33*	0.82
	Tense arousal	0.24	1.13	*0.37*	1.01
	Life engagement	0.14	1.03	*0.22*	0.99
	Rumination	0.24	1.05	*0.39*	0.93
**Testing sample**					
	Sleep quality	0.12	0.92	*0.16*	0.90
	Fatigue	0.21	0.94	*0.29*	0.89
	Good mood	0.28	0.94	*0.35*	0.89
	Tense arousal	0.23	1.14	*0.32*	1.07
	Life engagement	0.21	1.21	*0.25*	1.17
	Rumination	0.31	1.06	*0.40*	0.98

^a^GLM: general linear model.

^b^Italics indicate the highest *R*^2^ values for each outcome.

In the training sample, the penalized GLM performed best on all outcome measures. In the testing sample, the penalized GLM again performed the best followed by the random forest model.

Inspecting the most impactful predictors revealed that each model chose one or more unique participant identifiers as their primary source of information. Given that the rating scales restricted the participants’ answers to ranging between 1 and 5, we concluded that the models might have identified prototypical participant trajectories that were able to represent a larger proportion of the sample. Considering these results, it was unclear whether the sensor data would provide any meaningful information about the participants’ states on their own when the models were trained on between-participant data. Therefore, we continued the analysis by splitting the predictor set into the sensor data and the participant identifier.

Omitting the participant identifier resulted in a reduction in the predictive performance of both models. The *R*^2^ values for the random forests ranged from 0.01 (rumination and life engagement) to 0.05 (good mood) in the training sample and was approximately 0.01 in the testing sample for all outcomes. The *R*^2^ values for the penalized GLM ranged from 0.01 (sleep quality and rumination) to 0.03 (good mood and tense arousal) in the training sample and ranged from <0.01 (life engagement and rumination) to 0.01 (all other outcomes) in the testing sample.

Inspecting the importance of the predictors for the models using only the identifiers showed that the random forests and the penalized GLM chose the same participant identifiers to infer the outcomes except for tense arousal, which both models still inferred equally well. Such similarities were not found in the models without the person identifier.

On the basis of these results, it appeared that the sensor data obtained from the questionnaire were not suitable for training models that could replace self-reports.

### Personalized Models

After analyzing the data for the complete sample, we continued to train the models on the individual trajectory of each participant. Model performance was then evaluated by inferring the same participant’s responses on the last 5 mornings. We trained a total of 948 possible models for each algorithm. With the missing data now weighted against each individual model, 302 models could not be computed using random forests (sleep quality: 46/302, 15.2%; fatigue: 41/302, 13.6%; good mood: 48/302, 15.9%; tense arousal: 57/302, 18.9%; life engagement: 52/302, 17.2%; rumination: 58/302, 19.2%). Another 283 models could not be computed for the penalized GLM (sleep quality: 43/283, 15.2%; fatigue: 40/283, 14.1%; good mood: 44/283, 15.5%; tense arousal: 54/283, 19.1%; life engagement: 48/283, 17%; rumination: 54/283, 19.1%). Most (208/302, 75.5%) of the missing models were related to the same 38 participants with very low compliance in the morning questionnaire.

For all outcomes, the *R*^2^ values archived in the testing sample ranged from 0 to 1 for the random forests and penalized GLMs. For 75.3% (119/158) of the participants, the models showed an *R*^2^ value of ≥0.18. The descriptive statistics for the *R*^2^ distributions are presented in Table 2. Furthermore, we computed the accuracy criterion used in earlier studies [24,27]. The mean accuracy of the random forest models ranged from 41.37% (SD 30.86%) for life engagement to 51.38% (SD 32.05%) for good mood (sleep quality: mean 46.4%, SD 31.5%; fatigue: mean 42.4%, SD 29.29%; tense arousal: mean 41.88%, SD 29.62%; rumination: mean 45.06%, SD 33.78%). The mean accuracy for the penalized GLM ranged from 38.41% (SD 29.18%) for rumination to 48.19% (SD 31.48%) for good mood (sleep quality: mean 42.03%, SD 31.46%; fatigue: mean 43.92%, SD 29.87%; tense arousal: mean 41.19%, SD 28.58%; life engagement: mean 40.08%, SD 27.42%). On average, between 38.41% and 51.38% of the participants’ answers on the last 5 measurement occasions could be inferred from models trained on the individual sensor paradata of the first 13 measurements. Relating the accuracy to the mean compliance of participants during the last 5 mornings (mean 4.44, SD 0.82), this means that the models correctly inferred between 1.71 and 2.28 answers on average.

**Table 2:** Descriptive statistics for the distributions of *R^2^* values achieved on the testing set. Except for the tense arousal outcome, the penalized general linear model (GLM) performed better at inferring the last 5 mornings.

Outcome		Values, mean (SD)	Percentile 0	25th percentile	50th percentile	75th percentile	100th percentile
**Random forest**							
	Sleep quality	0.27 (0.28)	0	0.03	0.18	0.44	1
	Fatigue	0.32 (0.32)	0	0.05	0.21	0.54	1
	Good mood	0.28 (0.28)	0	0.05	0.2	0.43	1
	Tense arousal	0.34 (0.28)	0	0.1	0.3	0.52	1
	Life engagement	0.3 (0.3)	0	0.04	0.2	0.49	0.99
	Rumination	0.27 (0.29)	0	0.03	0.16	0.41	1
**Penalized GLM**							
	Sleep quality	0.35 (0.33)	0	0.05	0.26	0.63	1
	Fatigue	0.28 (0.27)	0	0.05	0.21	0.47	1
	Good mood	0.3 (0.28)	0	0.07	0.21	0.51	1
	Tense arousal	0.34 (0.3)	0	0.07	0.26	0.59	1
	Life engagement	0.33 (0.29)	0	0.07	0.26	0.5	1
	Rumination	0.31 (0.31)	0	0.07	0.18	0.48	1

### Further Analyses Regarding Features and Performance

To analyze the importance of the features, we summed the feature importance of all the individual models for each outcome. The algorithms predominantly used autocorrelations and partial autocorrelations from both sensors. Both algorithms tended to harness the higher-order autocorrelations, representing a correlation of values between 2 and 5 seconds rather than the lag-1 or lag-2 autocorrelations. The same held true for the partial autocorrelations. For the penalized GLM, the set of important features was complemented by the mean acceleration along the y-axis (vertical movement) for good mood, tense arousal, and rumination. In addition, the mean acceleration along the x-axis (horizontal movement) appeared to be important for inferring tense arousal. The random forest models used the measurement occasion to infer fatigue, tense arousal, and rumination. The algorithm also used the maximum acceleration along the y-axis, the maximum rotation around α (turning the phone to landscape mode), and the root mean square of successive differences of the rotation around α to infer good mood. It also used the SD of the acceleration along the z-axis (moving the phone back and forth) to infer life engagement. None of the algorithms showed a pattern that could be interpreted point-blank in terms of posture or exerted force.

We attempted to identify further similarities between the models by clustering the predictors based on their importance across all outcomes. To determine an empirical number of clusters, we conducted an LPA using the *mclust* package in R [62]. Inspecting the progression of the Bayesian information criterion (BIC) and the integrated completed likelihood for the random forests, both clustering algorithms favored a 1-cluster solution (BIC_EEI_=−3828.916; integrated completed likelihood_EEI_=−3828.916) regardless of the cluster parameterization [62]. We explored this further by testing the preferred solution of equally sized and distributed clusters (EII parameterization) with a bootstrap likelihood ratio test, testing successive cluster solutions for an improvement in model fit. This analysis also pointed toward a single-cluster solution (likelihood ratio test statistic_1 vs 2_=7.98; *P*=.54).

Contrary to the LPA for the random forests, the results for the penalized GLMs indicated a solution with multiple clusters. The BIC peaked between a solution with 5 and 7 clusters, with 5 clusters that were variable in size and rotation (VVE parameterized) as the favored outcome. However, the cluster solutions found for the penalized GLM aggregated the features into clusters of similar performance across all models instead of clusters targeted at individual outcomes. The cluster containing the features with the highest importance contained the most features (n=86) and, therefore, provided no advantage over the unclustered importance sums.

**Figure 2:**
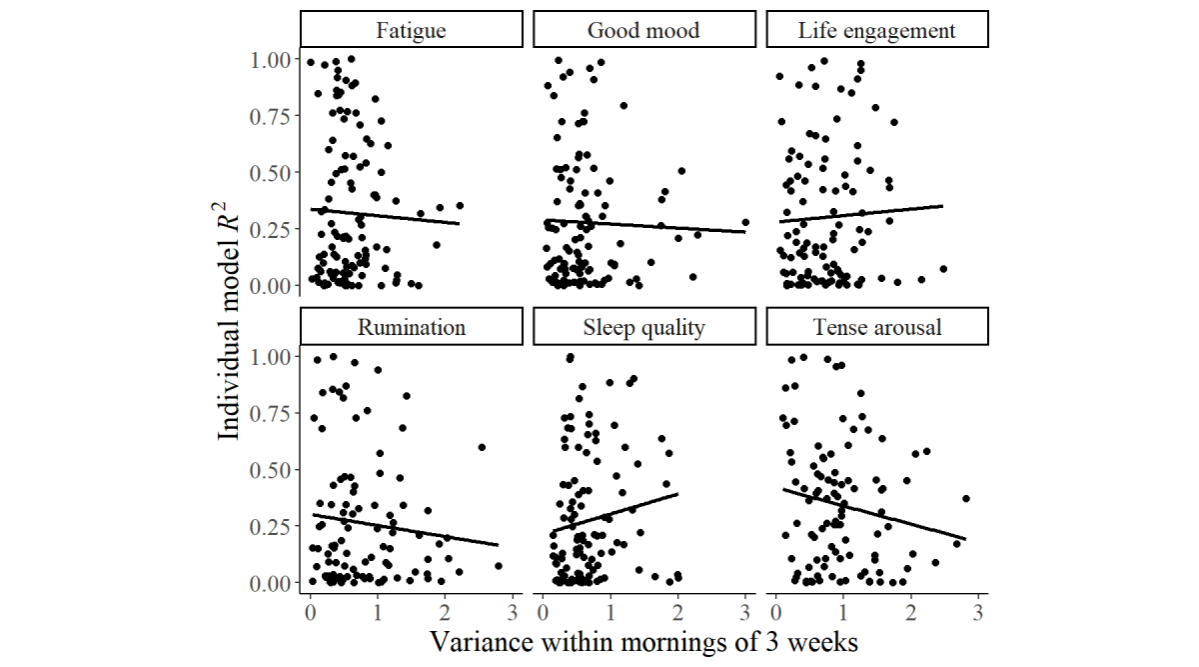
Scatterplots exploring the relationships between the variability of the outcomes across 3 weeks and the performance of the individual random forest models. Although some trends were depicted in the plot, we found no substantial correlations between variability and model performance.

Furthermore, we explored the relationships between the variability in the outcomes and model performance. All of the following correlations are Holm-corrected [63] for multiple comparisons. Correlation analyses (2-sided) between the square root–transformed individual models’ *R*^2^ results and the log-transformed variability of the self-report measures revealed no meaningful results for either algorithm. This was true for all outcomes as well as for the correlation between performance and variability within 3 weeks or only within the last week. The relationships between the (untransformed) model performance and the 3-week variance are depicted in Figure 2. Next, we included only results with *R*^2^≥0.01 in the analysis to explore whether more or less variability in the outcomes was correlated with models that explained ≥1% of the variance. This analysis also revealed no meaningful relationships.

We similarly analyzed the relationships between the questionnaires completed in the first 2 weeks and model performance. As we were not able to correct the skewed distribution of the compliance variable (most participants were very compliant), we calculated a 2-sided percentage bend correlation [64] as a nonparametric alternative to the Pearson correlation coefficient. This resulted in a negative relationship between compliance and model performance for the good mood outcome (*r*_percentage bend_(99)=−0.32; *P*=.02).

Finally, we visually inspected the outcome trajectories as well as the distributions of the sensor feature values between the 25% best-performing models and the 25% worst-performing models. Both visual analyses revealed no evident differences between the best- and worst-performing models.

## Discussion

### Principal Findings

This study examined the utility of sensor paradata that were passively collected while participants filled out EMA questionnaires to infer participants’ future self-reported mental states. Our results suggested that the sparse data collected only from movement-related sensors allowed us to infer participants’ self-reports on several outcomes related to mental well-being to some degree. For half of the participants, our models already performed well on all 6 outcomes. Using the accuracy criterion by Asselbergs et al [24], the mood-related personalized models in this study were able to replicate the lower boundary of previous work. Given that our models relied only on sparse data from the 2 movement-related sensors, this result is quite remarkable. This was even more remarkable given that the sample in our study was 5 times the size and consisted of a rather heterogeneous group of employees. This gives us confidence that further development in this area will refine the assessment to a point where passive sensing can actually replace self-reports.

Another result of our exploration was that our approach did not appear to be suitable for predictions trained on data from all participants, but it was suitable for modeling the states of the participants idiographically. The models resulting from training on the complete data set relied on the peculiarities of interindividual responses and started treating individual participant identifiers as the major source of information. Given the restricted ranges of Likert-type rating scales, deriving prototypes is a perfectly reasonable approach for capturing a good portion of the common variance between people, which could heuristically explain the results of the algorithms. Consequently, the models based on participant identifiers also worked, but the information from passive data had little to no impact on the final models. Another explanation could be that random variation between participants (eg, sensor readings that differed by device [44,65]) might have concealed relevant variance. This supports the point of Saeb et al [23] that models focusing on inferring future data from the same source should use personalized models. Consequently, we also advise future studies to clearly determine and disclose whether their goal is to classify participants or to infer participants’ future states with the trained models when choosing on which part of the data models should be trained and tested. As predicting the commonalities of a sample might most likely not be the goal of all studies using passive data and EMA, we suggest that the default of training between-participant models should be treated with caution when inferring participant mental states from sensor data instead of classifying passive data patterns. However, as demonstrated by Jacobson and Chung [25], such models can most likely be used to inform personalized models.

A final question we investigated was whether the working models could be traced back to a pattern of successful predictors that would be indicative of a certain outcome. In the related analyses, we found that, despite finding that the algorithms performed similarly, they treated the data differently. This was not too surprising as random forests rely on subsets based on chance and pick up on nonlinear relationships, whereas penalized GLMs rely on the complete data. For the random forest models, the LPA suggested a 1-cluster solution regarding the importance of features, but the LPA of the penalized GLMs showed no such classification tendencies. Instead, the analysis clustered indicators according to their impact with respect to all outcomes. Although both outcomes are semantically appealing, we would recommend that any interpretation be adapted with caution in future research on the relationships between sensor readings and psychometric outcomes.

We also did not find any intuitive relationships between model performance and the variability of the outcome data, participant compliance, the trajectory of the outcomes, or the distribution of the feature values. Although a singular negative relationship between the collected data and the model performance appeared to be significant, we assume this to be purely by chance despite the applied correction. As we asked about both mood subscales on the same questionnaire page, discovering an effect for only 1 subscale points to the difference in failed models between the 2 outcomes rather than a true correlation with compliance. Consequently, we would not expect to find this association between compliance and model performance in future studies.

### Potential Applications

We presented evidence that sparse movement sensor data collected in a privacy-friendly manner contain valuable information about the participant’s psychological state. However, so far it has not become explicit how these data might be used to significantly alleviate participant burden in EMA studies. At the moment, we envision 2 approaches that may foster participants’ compliance.

First, researchers might be able to omit arbitrary questionnaires after the training period. Reducing the length of the assessment comes with direct benefits for experienced burden and participant compliance [9]. Researchers would start with all relevant questionnaires in the survey and attempt to train personalized models on the relationship between sensor paradata features and scale values. Once those models are successfully established for the required number of participants and constructs, researchers could then omit arbitrary questionnaires from the survey and infer these missing responses by means of the trained models and the sensor paradata from the remaining questionnaires.

Second, researchers might be able to change the content of the survey during the assessment without compromising the completeness of the data set. Researchers would start with a subset of the relevant questionnaires. After training the personalized models on these data, one or more questionnaires could be replaced with other questionnaires. The trained models could then be used to infer the now unobserved constructs. Furthermore, if personalized models are trained on the added constructs, the resulting models could be used to infer the previously unobserved constructs. In this way, longer assessments could be shortened significantly to reduce the initial burden. Participants may also find the assessment less burdensome because of the regularly refreshed novelty.

The latter technique shares similarities with planned missingness designs [66] where certain items of a scale are randomized between participants. However, in contrast to planned missingness, the missing data are not inferred based on the entire sample’s answering behavior but through the biomarker provided by the individual participant. Given that further research can improve the accuracy of our method to the rates presented by LiKamWa et al [27], rotating questionnaires flanked by biomarkers might provide data with higher intrapersonal validity than planned missingness designs.

Both methods will still require an initial period to gather enough data to train the personalized models. In addition, we also assume that participants will be required to fill out questionnaires of a similar format after the training period, thus adhering to a semistandardized procedure. Other activities that might be enjoyable for the participants (eg, playing games) but require more physical activity might considerably affect the validity of the computed sensor features. However, these boundaries of applicability are subject to further systematic research as well as questions about the quantity of viable omissions and substitutions, the length of the required training period, and whether nonrectangular questionnaire formats (eg, slider bars and swipe choice) are suitable for training the personalized models.

### Limitations and Future Directions

One of the strongest points of this study is that we were able to demonstrate that models trained on paradata that can be collected while participants fill out a survey can be used to infer self-reported states over time. As demonstrated, this can be done completely unobtrusively alongside an already existing research project without a dedicated study setting focused on the exploration of passive data or special laboratory hardware. However, as this study is still exploratory, we want to emphasize some limitations that come with the degrees of freedom that the researchers will use when conducting research on passive data. As outlined in our theoretical introduction, changing the parameters may lead to different results in future examinations on the topic.

First, choosing the same resolution for the sensors (2 Hz) might be crucial for replicating our results. Smartphone sensors of movement values are usually able to report up to approximately 100 readings per second (100 Hz). Choosing a frequency that is appropriate for the task is an important part of analyzing sensor data [65]. In our study, these rather conservative values were chosen based on three deliberations targeting the applicability of the methods presented in this study:

- Sensor output should remain manageable by statistical software that is typically used in the social sciences. Sensor readings become big data very quickly, where traditional data analytic methods might still suffice, but data have to be handled via external databases. Data preparation then requires experience with software and programming that is not part of many medical or social science curricula. For comparison, the low-resolution sensor readings in this study already added up to approximately 7.5 million data points.
- Sensor data collection should be applicable to many different survey frameworks without the need for proprietary software. This perspective does not comprise the technical implementation regarding the application programming interface, which will be the same for all frameworks; rather, it is concerned with the space required to save the sensor streams. For example, in this study, we used the method of temporarily collecting the data per survey page in a long string of text in the web browser’s memory and then saved it to the survey’s database. Some survey frameworks might be limited even further when storing large chunks of text to a study variable.
- Sensor data collection should not interfere with the main questionnaire or device use. Collecting large chunks of data in the web browser’s memory may slow down the experience of using the questionnaire when older smartphones are used. As the training of the sensor models still relies on the self-reported outcomes, unnecessary dropout owing to inconvenience when completing the questionnaire should be avoided. In settings where dedicated apps are used, the sampling frequency could easily be increased.

Regarding the overall variation in the performance of the individual models, retrospectively, it would have been preferable to collect data on the specific devices that might have indicated some issues related to a family of devices or an operating system. Therefore, we recommend that future studies should at least record basic parameters such as screen resolution and the web browser that was used to rule out some device-specific issues.

Sensor data are also subject to the same *black box* problem that is immanent in any study in which experimenters and participants rarely meet each other. We do not know exactly what the participants were doing or experiencing while filling out the questionnaire. Consequently, a very poor-performing model might simply originate from a participant’s irregular behavior when filling out the questionnaire. However, contrary to the same issues with self-report data, combining sensors with pretrained classification algorithms might be fruitful for determining participants’ activity levels for each measurement and informing the models beyond the aggregated features [25,42].

Finally, although we were able to validate our method for a variety of psychological constructs, it is unclear whether this transitions to all possible time frames and topics. As demonstrated in our analyses, models will not learn when there are not sufficient data. This might limit the implementation of this personalized approach to studies that comprise contexts with at least 10 to 20 measurement points. Our study was also unable to answer questions about the underlying processes that determine a good model or features responsible for being a good marker regarding the outcomes we investigated. Consequently, the process between features and mental state itself remains largely unexplored. As we demonstrated in this study that data can be acquired almost effortlessly, we encourage future studies to examine and define the connections between the mind and the behavior we can record concurrently with the self-report.

### Conclusions

In our study, we demonstrated that a few unobtrusively collected movement sensor data are a capable foundation to train models that are able to infer a range of psychological constructs with sizable effects [67]. This study contributes to previous research by inspecting differences between models trained on between-person variance and personalized models. Our study showed that validating such models by inferring participants’ states in an independent data set can prove to be a fruitful approach. This applies even when relying on a privacy-friendly small amount of data that were only collected during measurement occasions of the self-report data collection. Finally, we expanded on previous work on combining personalized models with sensor data in the EMA context using a large and diverse sample. Despite basing our models only on movement-related sensors, we were able to replicate a comparable degree of accuracy. This worked best for outcomes that have already been demonstrated to work well in the literature (ie, mood) but also showed its potential regarding other outcomes (eg, life engagement).

Although sensor readings of physiological properties have a strong root in the history of medicine and psychology, the exploration of the relationships between everyday fluctuations in psychometric values and smartphone sensors is still in its infancy. Much more research on this topic will be required before it can become a valuable tool in mental health. We contributed to this goal by demonstrating that the accessible method of obtaining sparse movement sensor data that we outlined can be temporally interlinked with EMA studies without sacrificing accuracy compared with studies with specialized hardware, software, or a much larger data set. In the future, such methods might be able to provide valuable insights into the mental well-being of the participants while reducing burden in research and clinical application.

## Abbreviations

BIC: Bayesian information criterion
EMA: ecological momentary assessment
GLM: general linear model
LPA: latent profile analysis
